# Impaired Intestinal Mucosal Barrier upon Ischemia-Reperfusion: “Patching Holes in the Shield with a Simple Surgical Method”

**DOI:** 10.1155/2014/210901

**Published:** 2014-05-14

**Authors:** Olivér Rosero, Péter Ónody, Tibor Kovács, Dávid Molnár, Gábor Lotz, Szilárd Tóth, Zsolt Turóczi, András Fülöp, Dávid Garbaisz, László Harsányi, Attila Szijártó

**Affiliations:** ^1^1st Department of Surgery, Semmelweis University, Ulloi Street 78, Budapest 1082, Hungary; ^2^Department of Human Morphology and Developmental Biology, Semmelweis University, Tuzolto Street 58, Budapest 1094, Hungary; ^3^2nd Department of Pathology, Semmelweis University, Ulloi Street 93, Budapest 1091, Hungary; ^4^National Center of Epidemiology, Gyali Street 2-6, Budapest 1097, Hungary

## Abstract

Mesenteric ischemia-reperfusion (IR) is associated with impairment of the gut barrier function and the initiation of a proinflammatory cascade with life-threatening results. Therefore methods directed to ameliorate IR injury are of great importance. We aimed at describing the effects of postconditioning (PC) on the alterations of the intestinal mucosal function and the inflammatory response upon mesenteric IR. *Methods*. Male Wistar rats were gavaged with green fluorescent protein-expressing *E. coli* suspensions. Animals were randomized into three groups (*n* = 15), sham-operated, IR-, and PC-groups, and underwent 60 minutes of superior mesenteric artery occlusion, followed by 6 hours of reperfusion. Postconditioning was performed at the onset of reperfusion. Blood and tissue samples were taken at the end of reperfusion, for histological, bacteriological, and plasma examinations. *Results*. The PC-group presented a more favorable claudin-2, claudin-3, claudin-4, and zonula occludens-1 membrane expression profile, and significantly lower rates of bacterial translocation to distant organs and plasma D-lactate levels compared to the IR-group. Histopathological lesions, plasma I-FABP, IL-6, and TNF-**α** levels were significantly lower in the PC-group compared to the IR-group. *Conclusion*. The use of postconditioning improved the integrity of the intestinal mucosal barrier upon mesenteric IR, and thus reduced the incidence of bacterial translocation and development of a systemic inflammatory response.

## 1. Introduction

Intestinal ischemia is a severe condition, which is mostly the result of circulatory redistribution or the occlusion of the superior mesenteric artery (SMA). The occlusive form of acute mesenteric ischemia is mainly attributed to the embolization of the SMA, leaving the thrombosis of a preexistent atherosclerotic lesion to be the secondary cause of intestinal infarctions [1]. Although rapid surgical intervention and revascularization are essential, restoration of mesenteric perfusion inevitably induces the reperfusion injury of previously ischemic tissue [2]. Acute mesenteric ischemia-reperfusion (IR) is associated with high mortality rates due to the difficult diagnosis and the several concomitant complications [3, 4]. Among the most serious complications is the loss of gut barrier function, which predisposes to the egress of commensal flora from the intestinal lumen into the systemic circulation. Many studies have pointed out the link between bacterial translocation and postoperative septic complications and multiple organ dysfunction [5–8]. Despite the advances in diagnostics, treatment modalities, and intensive care, the mortality rates of acute mesenteric ischemia have remained as high as 60–90% [9].

The IR injury is mainly characterized by the release of free radicals at the early stage of reperfusion [10, 11], which is further aggravated by the overproduction of proinflammatory cytokines. Reactive oxygen species (ROS) react with the surrounding macromolecules leading to the peroxidation of membrane lipids, protein denaturation, and DNA damage [12]. Free radicals produced during reperfusion cause tissue breakdown and the disruption of tight junction protein components, leading to an increased intestinal permeability [13]. Grootjans et al. [14] reported, in a human model, that bowel IR induces the reduction of intestinal epithelial zonula occludens-1 (ZO-1) membrane expression, thus enhancing the translocation of endotoxin from the intestinal lumen into the circulation.

The protection of organs from IR injury has always been an immense challenge for surgeons. In case of acute mesenteric ischemia, the onset of the circulation impairment is impossible to predict, thus narrowing the therapeutic possibilities to options applied during the reperfusion period. Postconditioning has been stated to be a suitable method to ameliorate IR injury in various animal models [15–18]. It consists of a series of repeated brief cycles of vascular occlusion applied at the onset of reperfusion [19]. Our previous work demonstrated that the appropriate cycles of postconditioning are able to reduce the production of harmful free radicals by limiting the oxygen delivery into the ischemic area [20].

The present study was mainly designed to evaluate the effects of postconditioning on the intestinal mucosal permeability in a widely used rat model of mesenteric IR, which is capable of recreating events present during the occlusive entities of acute mesenteric ischemia [21, 22]. We used a green fluorescent protein- (GFP-) expressing* Escherichia coli* strain as a tracer to find causal association between bacteria present in the gut lumen and the extraintestinal sites. Furthermore subcellular epithelial alterations were investigated to confirm the impairment of intestinal barrier function found upon mesenteric IR.

## 2. Materials and Methods

### 2.1. Cultivation of GFP-Transfected* E. coli*

The experiments were performed with pGLO plasmid containing GFP-expressing* Escherichia coli* strain HB101 (NCAIM B01992; National Collection of Agriculture and Industrial Microorganisms, Budapest, Hungary). Bacteria were cultured on Luria-Bertani agar plate (LB, Difco recipe) with 0.2% L-arabinose (Sigma-Aldrich Inc., St. Louis, MO, USA) and 100 mg/L ampicillin (Sigma-Aldrich Inc.) at 37°C for 24–48 hours under aerobic conditions. The bacteria were cultured for the study to the density of 1 × 10^10^ colony-forming units per milliliter (CFU/mL). Bacterial concentration was determined by measuring the suspension turbidity with a spectrophotometer (optical density at 600 nm) and was verified by colony counting and standard serial dilutions techniques.

### 2.2. Animals

Inbred male Wistar rats, weighing 250–280 g, were used (Charles River Hungary Ltd., Budapest, Hungary). The experimental design was approved by the Animal Care Committee of the Semmelweis University (license number 22.1/2408/3/2011) and was performed in accordance with the US National Institute of Health guidelines (publication number 85-23, revised 1996; Bethesda, Maryland). Animals were kept under specific pathogen-free conditions at 22–24°C. They were fed with commercial pellets and water* ad libitum*. Twelve hours prior to operation only water was given and 1 mL of 1 × 10^10^ CFU/mL GFP-expressing* E. coli* suspension was administered to each animal via oroduodenal catheterization [21]. Each experiment started at the same time of the day to avoid the effects of circadian rhythm.

### 2.3. Operative Procedure

The animals (*n* = 45; 15 in each group, according to study design) were anaesthetized using an intraperitoneal injection of ketamine (75 mg/kg) and xylazine (7.5 mg/kg). They were then placed in supine position on a heating pad to keep their body temperatures between 36.5°C and 37.5°C, monitored by a rectal thermometer (Homeothermic Blanket Control Unit, Harvard Apparatus, Holliston, MA, USA).

A polyethylene catheter was inserted into the right jugular vein in order to maintain anesthesia and to compensate intraoperative fluid loss by the administration of physiological saline solution (3 mL/bwkg/h). Median laparotomy was performed and the SMA was identified. Mesenteric warm ischemia was induced by clamping the SMA for 60 minutes, using an atraumatic microvascular clip (Harvard Apparatus). Mesenteric ischemia was followed by 6 hours of reperfusion. During the IR period, the animal's abdomen was covered with a plastic blanket to prevent fluid loss via evaporation. In the postconditioned-group (PC), after the ischemic interval, postconditioning was performed by 6 alternating cycles of opening and closing the microvascular clip placed on the SMA, each cycle lasting 10 seconds [20]. After 6 hours of reperfusion the animals were sacrificed by exsanguination via right ventricular puncture. Collected blood was centrifuged (3000 rpm for 2 × 10 minutes, at room temperature); plasma was snap-frozen in liquid nitrogen and stored at −80°C until further analysis. Under aseptic conditions mesenteric lymph node (MLN), spleen, liver, lung, and kidney biopsies were obtained. Histological samples were taken from the middle part of the duodenum, the jejunum, and the ileum: 10 mm long slices were placed in 4% neutral-buffered formalin and further 10 mm long adjacent parts were snap-frozen in liquid nitrogen. The remnant mucosal mass was homogenized, snap-frozen, and stored at −80°C until further analysis.

### 2.4. Experimental Groups

The animals were randomly divided into three groups (*n* = 15 in each) as follows.

1. * Sham*-operated group: after opening the abdomen the SMA was dissected, but ischemia was not induced. After 7 hours of observation the experiment was terminated and samples were taken.
2. *IR*: in the “ischemia-reperfusion” group animals underwent 60 minutes of SMA occlusion, followed by 6 hours of reperfusion.
3. * PC*: in the “postconditioning” group 6 alternating cycles of reperfusion-reocclusion, lasting 10 seconds each, were performed at the onset of reperfusion.

### 2.5. Microbiological Analysis

For the quantitative detection of the translocated GFP-expressing* E. coli* in the extraintestinal sites, tissue samples weighing 0.1 g were homogenized in 1 mL of sterile physiological saline and 5 decimal dilution series were made from each sample. 200 *μ*L aliquots of the homogenate and the dilutions were cultured in triplicate on Luria-Bertani agar media supplemented with L-arabinose and ampicillin for detection of GFP-expressing* E. coli*. The plates were examined after 24–48-hour incubation at 37°C in order to count the colony-forming units (CFU) bacteria per gram of tissue. The colony counting was assessed under 312 nm wavelength UV light. The number of viable colonies was counted in each dilution and multiplied by the appropriate dilution factor.

### 2.6. Histopathological Analysis

The excised duodenal, jejunal, and ileal samples were fixed in 4% neutral-buffered formalin for 24 hours, dehydrated, and embedded in paraffin. Three *μ*m thick sections were stained with hematoxylin and eosin (HE). Histopathological changes were evaluated using Chiu's score, which is utilized to describe and quantify the degree of mucosal injury associated with IR injury of the intestine [23]. In grade 0 villi are normal, in grade 1 there is a development of a subepithelial space usually at the apex of the villi with capillary congestion, in grade 2 the subepithelial space extends with moderate lifting of the lamina propria, in grade 3 a massive epithelial lifting is present down the sides of the villi, possibly with a few denuded tips, in grade 4 there are denuded villi with exposed lamina propria and dilated capillaries, possibly with increased cellularity of the lamina propria, and in grade 5 there is digestion and disintegration of the lamina propria, hemorrhage, and ulceration. The histological evaluation was performed by two independent pathologists who were blinded to the applied treatment and groups. All sections were studied using light microscopy (Olympus BX 50 microscope, Olympus Micro Bright Field, Williston, VT, USA).

### 2.7. Claudin-2, Claudin-3, Claudin-4, and Zonula Occludens-1 Immunohistochemistry

Three *μ*m thick cross sections were prepared from formalin fixed and paraffin embedded duodenum, jejunum, and ileum samples. Samples were deparaffinized and rehydrated, and the endogenous peroxidase activity was quenched with 0.6% H_2_O_2_ in methanol. Nonspecific binding sites were blocked with bovine serum albumin for 30 minutes. Primary rabbit anti-rat antibodies against claudin-2, claudin-3, claudin-4, and zonula occludens-1 (51-6100; 34-1700; 36-4800; and 40-2300, resp.; Invitrogen, Carlsbad, CA, USA) were applied in 1 : 50, 1 : 100, 1 : 100, and 1 : 125 dilutions, respectively, and were incubated overnight at 4°C. After washing, sections were incubated in biotinylated anti-rabbit secondary antibody (Vector Laboratories, Burlingame, CA, USA) diluted to 1 : 200 in blocking plasma for 30 min. Binding sites were demonstrated with avidin-biotin-peroxidase complex (Vectstain Elite ABC kit, Vector Laboratories) and colour precipitation was developed with DAB substrate kit (Vector Laboratories). Slides were washed and counterstained with Gill's hematoxylin (Accustain, Sigma-Aldrich, St. Louis, MO, USA). As a negative control, sections were simultaneously stained while omitting the primary antibodies. Each specimen was semiquantitatively scored by two independent pathologists blinded to the treatment and groups. Ten high-powered fields were randomly selected and each individual villus was graded. To focus on the alterations of the intercellular tight junction proteins membrane expression, reactions were scored positive where linear membrane staining was seen. The immunoreactivity was evaluated by the percentage of intestinal epithelial cells with positive membrane staining using the following criteria: 0: absence of membrane staining; 1: fewer than 20% of intestinal epithelial cells with membrane staining; 2: 21% to 40% of intestinal epithelial cells with membrane staining; 3: 41% to 60% of intestinal epithelial cells with membrane staining; 4: 61 to 80% of intestinal epithelial cells with membrane staining; 5: 81% to 100% of intestinal epithelial cells with membrane staining [24].

### 2.8. Measurement of ROS

A chemiluminescent assay was used to measure reactive oxygen species present in the small intestine mucosal homogenates. Chemiluminescence was detected in an H_2_O_2_/OH^−^-luminol-microperoxidase system, using a Lumat LB 9051 luminometer (Berthold Technologies GmbH, Bad Wildbad, Germany) [25]. Light intensity, given in relative light unit percent of the background (RLU%), was proportional to the concentration of free radical compounds in the sample. Luminol, microperoxidase, and hydrogen peroxide were obtained from Sigma-Aldrich Inc. (St. Louis, MO, USA); other chemical reagents were purchased from Reanal Chemical Co. (Budapest, Hungary).

### 2.9. Measurement of Plasma Intestinal Fatty Acid Binding Protein, Interleukin-6, Tumor Necrosis Factor-*α*, and D-lactate

Plasma intestinal fatty acid binding protein (I-FABP), IL-6, and TNF-*α* levels were measured using commercially available enzyme immunoassay kits from TSZ ELISA (TSZ Scientific, Framingham, MA, USA) and Quantikine Rat IL-6 and TNF-*α* immunoassay kit (R&D Systems, Minneapolis, MN, USA). Plasma D-lactate levels were measured using commercially available colorimetric assay kit from Sigma-Aldrich Inc. following manufacturer's instructions. Absorbances were measured at 450 nm.

### 2.10. Data Collection and Statistical Analysis

Results were expressed as means ± standard deviation (SD). Normality of all data was verified by the Kolmogorov-Smirnov test. Variance equality between groups was tested by Levene's statistical analysis. One-way ANOVA was used to detect differences between groups. Post hoc comparisons were made using Scheffe's test. The difference in the histopathological grades and staining intensity scores were statistically evaluated using nonparametric Kruskal-Wallis test. Post hoc analyses for pairwise comparisons between groups were performed using Mann-Whitney *U* test. Statistical evaluation for proportional comparisons of tissue cultures was made using the Chi-square test. A value of *P* < 0.05 was considered as statistically significant difference. Statistical analyses were performed using SPSS Statistics 20.0 software (IBM Corporation, Armonk, NY, USA).

## 3. Results

### 3.1. Microbiological Analysis

**Figure 1:**
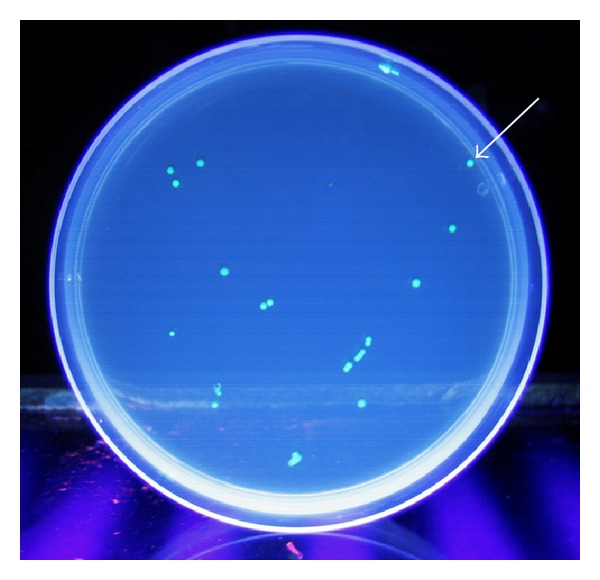
Colony counting method on a Petri plate with Luria-Bertani agar media, under UV light. The plate represents the result of culturing 200 *μ*L aliquots prepared from 0.1 g liver sample homogenized with 1 mL sterile physiological saline solution. Twenty-five (arrow) green fluorescent protein-expressing* Escherichia coli* colony-forming units (CFU) can be identified, which correspond to a 1250 CFU/g concentration of bacteria in the examined sample.

Tissue homogenates of the sham-operated group presented no bacterial colonization. The groups subjected to IR, however, had a variable range of detectable GFP-expressing* E. coli* in the culture plates. The use of postconditioning not only decreased significantly the incidence of bacterial translocation to extraintestinal sites (MLN, spleen, liver, lung, and kidney) induced by IR, but also diminished markedly the concentration of translocated bacteria (Tables 1 and 2 and Figure 1).

### 3.2. Histopathological Analysis

**Figure 2:**
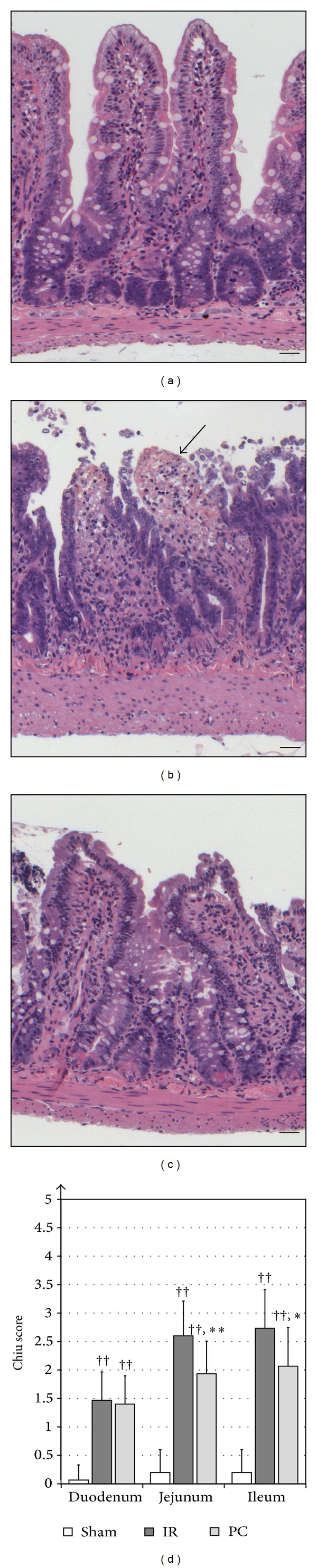
Representative hematoxylin-eosin stained histological samples of the jejunum in the three experimental groups. The jejunal section of the sham-operated group (a) represents an unimpaired intestinal villi structure. The ischemia-reperfusion- (IR-) group (b) shows several denudated villi with partial digestion, as shown by the black arrow. The intestinal villi also showed massive epithelial lifting, which extended down the sides of the villi in some cases. Increased cellularity of the lamina propria was also detected. The postconditioned group (c) exhibits a more benign picture, with mild to moderate epithelial lifting. Scale bars: 50 *μ*m. The bar graph (d) shows the Chiu scores of the three segments of the small intestine in the different experimental groups. Sixty minutes of superior mesenteric artery occlusion and six hours of reperfusion caused significant histopathological changes in the three segments of the small intestine compared with the sham-operated group. The use of postconditioning at the onset of the reperfusion significantly ameliorated the histologic injury observed in the jejunum and ileum segments of the IR-group. Results are expressed as means ± SD (*n* = 15 in each group). (††): *P* < 0.01 versus sham-group; (∗): *P* < 0.05 versus IR-group; (∗∗): *P* < 0.01 versus IR-group.

Regarding the histologic samples stained with hematoxylin and eosin, in the sham-group no remarkable morphologic changes were observed in the three small bowel segments. In contrast, relevant changes were observed in the groups subjected to IR when compared to the sham-operated group. No statistically significant differences were observed in the duodenum samples between the IR- and the PC-group (*P* = 0.72). The use of postconditioning mitigated the severity of IR injury related to the jejunum and ileum segments of the bowel. Using Chiu score to quantify the lesions, the intensity of injury concerning the jejunum (*P* < 0.01 versus IR) and ileum (*P* < 0.05 versus IR) of the PC-group was significantly lower compared with the IR-group (Figure 2).

### 3.3. Immunohistochemical Analysis of Tight Junction Proteins

**Figure 3:**
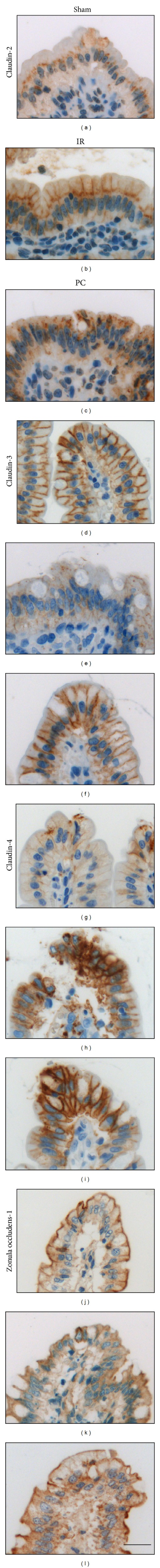
Representative photographs of claudin-2, claudin-3, claudin-4, and zonula occludens-1 immunostained in longitudinal jejunal sections of the three experimental groups. Claudin-2 immunoreaction in the sham-operated group (a) was low and was mainly found in the cytoplasm of the intestinal epithelial cells. Claudin-2 expression in the groups exposed to ischemia-reperfusion was substantially higher and was principally found in the lateral membrane of the epithelial cells (b, c). The sham-operated group presented strong membrane claudin-3 immunostaining (d), which was remarkably decreased in the ischemia-reperfusion- (IR-) group (e), but not in the postconditioned- (PC-) group (f). Minimal claudin-4 immunostaining was observed in the jejunal samples of the sham-operated animals (g). The two groups challenged with 60 minutes of mesenteric ischemia and 6 hours of reperfusion (h, i) showed high membrane claudin-4 expression, which was limited to the tips of the villi. Zonula occludens-1 was strongly immunostained in the jejunal samples of the sham-operated animals (j). Following small intestinal ischemia-reperfusion, intestinal epithelial cell with zonula occludens-1 membrane immunostaining notably decreased in the IR-group (k); in the PC-group minimal loss of zonula occludens-1 membrane immunostaining was detected (l). Each photograph was taken with the same magnification. Scale bar: 20 *μ*m.

In the samples of the sham-operated animals, claudin-2 was weakly stained in all the three small intestinal segments. A mainly granular cytoplasmic positivity was observed for claudin-2, principally in the crypts of the villi with sparse membrane positivity. The two groups exposed to IR presented higher claudin-2 membrane expression, with a marked accumulation in the lateral membranes of the epithelial cells. Jejunal and ileal segments of the IR-group showed significantly major claudin-2 membrane positivity compared with the samples of the postconditioned group. Claudin-3 gave a strong membranous reaction along the crypt-villus axis of the sham-operated group, showing no remarkable gradient in either of the intestinal segments. Mesenteric IR caused a dramatic decrease of claudin-3 expression in the epithelial cells of the jejunum and ileum. Postconditioning significantly attenuated the loss of claudin-3 membrane staining in the two above-mentioned small intestinal segments. Claudin-4 staining was poorly detected in all the examined samples of the sham-operated group and was mainly found in the cytoplasm of the epithelial cells at the tips of the villi. Ischemia-reperfusion substantially induced claudin-4 expression in all the small intestinal segments and was restricted to the membrane of the epithelial cells located at the tips of the villi. The percentage of intestinal epithelial cells with claudin-4 membrane expression did not differ significantly between the IR- and postconditioned-groups. ZO-1 was detected as membranous staining principally on the lateral and apical surfaces of the epithelial cells. ZO-1 was uniformly observed in the duodenum, jejunum, and ileum of the sham-operated group. Following mesenteric IR samples taken from the jejunum and ileum displayed significantly lower ZO-1 membrane expression. However, the jejunal and ileal sections of the postconditioned group presented significantly more intestinal epithelial cells with ZO-1 membrane staining compared with the IR-group (Table 3 and Figure 3).

### 3.4. Intestinal Mucosal ROS Stress

Chemiluminescent intensity, which is proportional to the concentration of ROS in the examined sample, was significantly increased in the two groups challenged with mesenteric IR in comparison to the sham-operated group. The use of postconditioning was able to significantly mitigate the production of ROS in the jejunum and the ileum. This effect was tendentiously seen when comparing the duodenum homogenates of the IR- and the PC-groups (*P* = 0.091) (Table 4).

### 3.5. Changes in the Plasma I-FABP, D-lactate, IL-6, and TNF-*α* Levels

The plasma I-FABP levels rose significantly upon mesenteric IR, which was significantly attenuated in the PC-group. There was a significant elevation in the plasma D-lactate levels detected in the groups exposed to IR, when compared with the sham-operated group. The level of plasma D-lactate measured in the PC-group was significantly lower than in the IR-group. The plasma IL-6 and TNF-*α* levels of the two groups subjected to IR were found to be substantially elevated in comparison to the levels of the sham-operated group. Levels of both proinflammatory cytokines were significantly lower in the PC-group, when compared with the IR-group (Table 5).

## 4. Discussion

Acute mesenteric ischemia is a life-threatening condition requiring urgent surgical intervention. Paradoxically the lifesaving revascularization further aggravates the small intestinal tissue damage causing the so-called reperfusion injury. It has been claimed that most of the intestinal tissue damage occurs after the reinitiation of tissue perfusion [2], which underlines the importance of therapies, aiming to attenuate reperfusion injury. Performing postconditioning with the use of an atraumatic, curved vascular clamp, by short alternating cycles of declamping and reclamping, at the time of sustained revascularization, can be a simple and clinically applicable method to mitigate the degree of IR injury [18, 26].

The present study was elaborated to examine the effects of postconditioning on the mucosal IR injury of the small intestine in a model of 60 minutes of SMA occlusion and 6 hours of reperfusion. The 60 minutes of ischemia was set as a period long enough to induce measurable and reproducible damage of the intestinal mucosa [27]; furthermore, the 6 hours of reperfusion were adopted as the shortest interval for significant detectable bacterial colonization of the extraintestinal tissues [28]. Postconditioning was applied at the onset of the reperfusion and the algorithm was chosen in line with the results of a previously published comparative study, which demonstrates the superior effectivity of short and dynamic cycles of postconditioning [20].

Mesenteric IR caused remarkable intestinal mucosal injury, mainly in segments of the jejunum and the ileum, generating extensive epithelial detachment from the lamina propria and few denudated villi. The plasma I-FABP levels were assessed as a sensitive serological marker of intestinal mucosal injury [29–31]. I-FABP is a cytosolic protein mainly synthetized by enterocytes situated at the tips of the villi and is released into the bloodstream in cases of enterocyte damage [32]. In the current study, histopathologic changes induced by IR were accompanied by a substantial elevation of the plasma I-FABP levels, corroborating the injury of the intestinal mucosal layer. According to the histological observations, postconditioning reduced the severity of intestinal mucosal injury and the concomitant elevation of plasma I-FABP level.

The intact intestinal unicellular epithelium serves as the only physical barrier between the luminal bacterial milieu and the sterile bloodstream [33], allowing the absorption of nutrients at the same time. This duality of functions of the enterocytes is achieved by the fine regulation of the paracellular pathway, which is mediated mainly by tight junctions located at the apical end of the cells. Claudins are transmembrane protein structures that are connected to the actin cytoskeleton through zonula occludens scaffold proteins and play a fundamental role in the formation of tight junction strands [34]. Epithelial cells can express multiple pore forming and barrier tightening claudins in a tissue specific manner, thus creating distinctive semipermeable barriers [35]. The expression of the different types of claudins and thus the paracellular permeability is dynamically regulated by various extracellular stimuli like cytokines, oxidative stress, or luminal bacteria and their products [36–39]. To characterize the integrity of the epithelial tight junctions, claudin-2, claudin-3, claudin-4, and ZO-1 proteins were immunostained. These tight junction components have previously been reported to be present in the rat small intestine [38, 40, 41]. The expression of claudin-2 has been associated with an increase of the paracellular permeability, through the formation of paracellular channels [42–44]. In contrast, claudin-3 and claudin-4 are categorized as “sealing claudins,” as their presence is related to an increase in the barrier tightening function [37, 45, 46]. Furthermore, claudin-4 has been claimed to play an important role in cell migration required for the physiological recovery of the intestinal epithelial layer [38]. Cytoskeletal regulation of tight junctions is achieved by anchoring the transmembrane proteins to the actin cytoskeleton, which is mediated by zonula occludens-1 [47]. Moreover, the downregulation of ZO-1 has been associated with a considerable increase of the intestinal permeability [48]. The immunohistochemical analysis revealed an increased redistribution of claudin-2 and claudin-4 from the cytoplasm to the intercellular membrane compartment, while claudin-3 and ZO-1 membrane expression decreased upon IR; these alterations are in accordance with reported tendencies [38, 49–51]. The changes detected in the IR-group were mitigated by the use of postconditioning, suggesting a less permeable tight junction protein profile, similar to the pattern observed in the sham-operated group. The increased expression of claudin-4 at the tips of the villi in both groups exposed to IR may be explained by the role of claudin-4 in the restoration of the damaged epithelial layer, as described by Frank [52] and Inoue et al. [38].

The decrease of “sealing” tight junction proteins and the increase of pore forming claudins indicate a perturbed epithelial layer [53]. The plasma levels of the bacterial fermentation product D-lactate have been described to be an effective tool to assess mucosal barrier integrity [31]. Murray et al. [54] reported an elevation of plasma D-lactate level following gut IR. This change was attributed to the loss of normal host defenses against bacterial overgrowth and the impairment of the mucosal barrier function, which enables the increased volume of bacteria and their metabolic products to egress into the circulation. In the present study, IR was associated with a significant elevation of plasma D-lactate levels, which is in agreement with literature data [55]. The application of postconditioning resulted in remarkably lower plasma D-lactate levels, suggesting a lesser extent of mucosal barrier damage [56]. Disruption of the intestinal barrier function leads to increased mucosal permeability allowing the passage of viable resident bacteria or its components from the gastrointestinal tract to extraintestinal tissues [33]. This phenomenon has been termed “bacterial translocation” [57]. To further examine the intestinal mucosal barrier function, bacterial translocation rates into the mesenteric lymph nodes, spleen, liver, lung, and kidneys were determined. In order to confirm that isolated bacteria were from intestinal origin, a definitive marker had to be established. Therefore, green fluorescent reporter protein-expressing* E. coli* was administered orally to the rats 12 hours prior to surgery. Mesenteric IR insult caused a significant incidence of bacterial translocation to the extraintestinal tissues, especially to the mesenteric lymph nodes and spleen. According to Berg [58], these organs are the first “station” of bacteria translocated from the gastrointestinal tract and frequently present higher concentrations of bacteria [59–61]. Postconditioning significantly decreased the incidence of bacterial translocation and the concentration of GFP-expressing* E. coli* in the positive samples, further corroborating the effectivity of postconditioning in the preservation of the mucosal barrier function.

The detrimental effects of mesenteric IR on the intestinal mucosal function are mainly mediated by the overproduction of free radicals [2, 62] and the initiation of proinflammatory cascades [63]. Oxidative stress has been associated with the disruption of tight junctions by several mechanisms such as the elevation of oxidized membrane lipids that alter the localization of tight junction proteins and the direct alteration of claudins extracellular redox sensitive motifs [37–39, 64]. Postconditioning has been postulated to downregulate the generation and release of ROS in models of intestinal IR [20, 65]. The importance of intervening at the onset of the reperfusion is underlined by the recognition that in the first minutes of the reperfusion there is a substantial burst of oxygen-derived free radicals. Limiting the “oxygen load” at this stage can influence the free radical burden during the latter phase of the reperfusion [10, 20, 66, 67]. According to the chemiluminescence assay, IR generated an elevation of ROS in the mucosal homogenates of the small intestine, which was successfully ameliorated by the use of postconditioning.

The release of free radicals during reperfusion has been shown to activate NF-*κ*B-dependent expression of cytokines that play a pivotal role in the pathophysiology of intestinal IR and generalization of local damage [68, 69]. In addition, proinflammatory cytokines have been demonstrated to be capable of increasing tight junction permeability, by inducing the rearrangement of the different tight junction proteins [36]. TNF-*α* and IL-6 have been claimed to impair intestinal tight junction function by the induction of claudin-2 and downregulation of ZO-1 expression [70, 71]. The proinflammatory cytokines TNF-*α* and IL-6 were markedly higher in the groups exposed to IR but were significantly lower in the PC-group.

Our study has some limitations. We used a sublethal model of intestinal IR and thus associations between the use of postconditioning and mortality rates cannot be made. Mucosal barrier permeability changes were not directly measured. Furthermore, the current study is limited to examine the alterations of four tight junction proteins. Direct quantitative assessments for tight junctions mRNA and protein expression were not carried out. These are areas of future research.

## 5. Conclusion

This study demonstrates the detrimental effects of mesenteric IR on the intestinal mucosal barrier function through the generation of ROS and the alteration of several tight junction components. The study describes the impact of intervening in the early period of reperfusion applying a surgical method called postconditioning. Postconditioning decreased the production of mucosal ROS and was associated with a milder histopathological picture. Plasma I-FABP levels also confirmed the lower degree of mucosal injury. Decreased plasma D-lactate levels and the distribution of epithelial tight junction proteins claudin-2, claudin-3, claudin-4, and ZO-1 justified the reduced rates of bacterial translocation, suggesting an improved integrity of the intestinal mucosal barrier function in the postconditioned group. Postconditioning seems to be an efficient method to timely and accurately target the site of ROS production and action, thus ameliorating IR injury.

## Figures and Tables

**Table 1:** Incidence of green fluorescent protein-expressing *Escherichia coli* translocation.

Experimental group	MLN	Spleen	Liver	Lung	Kidney
Sham	0/15	0/15	0/15	0/15	0/15
IR	14/15	13/15	12/15	10/15	9/15
	(93%)	(86%)	(80%)	(67%)	(60%)
PC	6/15**	5/15**	5/15*	3/15*	3/15*
	(40%)	(33%)	(33%)	(20%)	(20%)

Results show the incidence of green fluorescent protein-expressing *Escherichia coli* isolated from the harvested tissue homogenates. The proportional comparisons of tissue cultures were made using the Chi-square test. (∗): *P* < 0.05 versus IR-group; (∗∗): *P* < 0.01 versus IR-group.

IR: ischemia-reperfusion; PC: postconditioning; MLN: mesenteric lymph node.

**Table 2:** Concentration of green fluorescent protein-expressing *Escherichia coli* isolated from the extraintestinal tissues.

Experimental group	MLN	Spleen	Liver	Lung	Kidney
Sham	—	—	—	—	—
IR	1498 ± 76	1362 ± 92	1306 ± 89	1113 ± 128	1080 ± 101
PC	1197 ± 60**	1140 ± 77**	1068 ± 71**	930 ± 25*	910 ± 33*

Results are expressed as colony-forming unit per gram (CFU/g) and are means ± SD; *n* = 15 in each group. One-way analysis of variance was used for comparison of all groups. The difference between the groups by Scheffe's post hoc test: (∗): *P* < 0.05 versus IR-group; (∗∗): *P* < 0.01 versus IR-group.

IR: ischemia-reperfusion; PC: postconditioning; MLN: mesenteric lymph node.

**Table 3:** Semiquantitative analysis of intestinal epithelial tight junction components according to immunostained sections.

Assessed intestinal segment	Experimental group	Claudin-2	Claudin-3	Claudin-4	Zonula occludens-1
Duodenum	Sham	0.06 ± 0.2	5	0.2 ± 0.4	3.73 ± 0.4
	IR	2.27 ± 0.4^††^	4.67 ± 0.4^†^	1.4 ± 0.5^††^	3.47 ± 0.5
	PC	2.06 ± 0.2^††^	4.7 ± 0.4^†^	1.13 ± 0.3^††^	3.4 ± 0.5
Jejunum	Sham	0.2 ± 0.4	4.87 ± 0.3	0.2 ± 0.4	3.53 ± 0.5
	IR	3.47 ± 0.5^††^	1.33 ± 0.5^††^	2.2 ± 0.4^††^	1.33 ± 0.6^††^
	PC	2.93 ± 0.6^††,∗^	4.6 ± 0.5**	1.8 ± 0.6^††^	3 ± 0.5^†,∗∗^
Ileum	Sham	0.13 ± 0.3	4.93 ± 0.2	0.27 ± 0.4	3.73 ± .04
	IR	3.6 ± 0.5^††^	1.2 ± 0.4^††^	2.53 ± 0.5^††^	1.13 ± 0.3^††^
	PC	3.06 ± 0.5^††,∗^	4 ± 0.7^††,∗∗^	2.2 ± 0.4^††^	2.4 ± 0.5^††,∗∗^

Results are expressed as means ± SD; *n* = 15 in each group. Nonparametric Kruskal-Wallis test was used for comparison of all groups. The difference between the groups by Mann-Whitney *U* test: (†): *P* < 0.05 versus sham-group; (††): *P* < 0.01 versus sham-group; (∗): *P* < 0.05 versus IR-group; (∗∗): *P* < 0.01 versus IR-group.

IR: ischemia-reperfusion; PC: postconditioning.

**Table 4:** Small intestine mucosal ROS concentration of the three experimental groups measured by luminol-dependent chemiluminescence response.

Measured parameter		Sham	IR	PC
ROS-induced luminol chemiluminescence (RLU%)	Duodenum	243 ± 9	288 ± 10^††^	280 ± 9^††^
	Jejunum	250 ± 12	326 ± 17^††^	300 ± 17^††,∗∗^
	Ileum	265 ± 14	360 ± 19^††^	330 ± 13^††,∗∗^

Small intestine mucosal ROS content induces luminol chemiluminescence. The light intensity is proportional to the concentration of ROS in the sample. Results are expressed as means ± SD; *n* = 15 in each group. One-way analysis of variance was used for comparison of all groups. The difference between the groups by Scheffe's post hoc test: (††): *P* < 0.01 versus sham-group; (∗∗): *P* < 0.01 versus IR-group.

IR: ischemia-reperfusion; PC: postconditioning; RLU: relative light unit, an arbitrary unit of chemiluminescence measurement.

**Table 5:** Data measured from plasma samples of the three experimental groups.

Measured parameter	Sham	IR	PC
I-FABP (ng/mL)	7.5 ± 0.5	86.3 ± 3.7^††^	66.8 ± 5.5^††,∗∗^
D-lactate (ng/uL)	11.5 ± 0.5	23.5 ± 2.4^††^	15.8 ± 4.5^†,∗∗^
IL-6 (pg/mL)	92.4 ± 8.1	417.5 ± 47^††^	186.6 ± 43.4^††,∗∗^
TNF-*α* (pg/mL)	14.2 ± 3.2	59.3 ± 3.2^††^	41.2 ± 7.8^††,∗∗^

Results are expressed as means ± SD; *n* = 15 in each group. One-way analysis of variance was used for comparison of all groups. The difference between the groups by Scheffe's post hoc test: (†): *P* < 0.05 versus sham-group; (††): *P* < 0.01 versus sham-group; (∗∗): *P* < 0.01 versus IR-group.

I-FABP: intestinal fatty acid binding protein; IR: ischemia-reperfusion; PC: postconditioning.
